# 

**Published:** 1907-12-28

**Authors:** 


### METROPOLITAN HOSPITAL SUNDAY FUND.

With reference to the report of the annual meeting of the constituents of this fund on the 17th instant, which appeared on page 327 of our issue last week, the name of the Royal Commission on Vivisection was wrongly given in the first edition of our issue as the Anti-Vivisection Committee. The error was corrected in the second edition of last week's issue.

THE HOSPITAL *December* 28, 1907.

*Name* ........................................................................

*Address* ........................................................................

........................................................................

This Coupon must accompany manuscript or contributions intended for The Hospital.

